# Rare Manifestation of a Rare Disease, Acute Liver Failure in Adult Onset Still's Disease: Dramatic Response to Methylprednisolone Pulse Therapy—A Case Report and Review

**DOI:** 10.1155/2014/375035

**Published:** 2014-06-04

**Authors:** Nalini Valluru, Venkata S. Tammana, Michael Windham, Eyasu Mekonen, Rehana Begum, Andrew Sanderson

**Affiliations:** ^1^Department of Internal Medicine, Division of Gastroenterology and Hepatology, Virginia Commonwealth University, West Hospital, 14th Floor, 1200 East Broad Street, P.O. Box 980341, Richmond, VA 23298, USA; ^2^Department of Internal Medicine, Division of Gastroenterology and Hepatology, Howard University Hospital, 2041 Georgia Avenue NW, Washington, DC 20060, USA

## Abstract

Adult onset Still's disease (AOSD) is a rare systemic inflammatory disorder of unknown etiology. It is characterized by daily fevers, arthralgias or arthritis, typical skin rash, and leukocytosis. Hepatic involvement is frequently observed in the course of AOSD with mildly elevated transaminases and/or hepatomegaly. Fulminant hepatic failure, occasionally requiring urgent liver transplantation, is a rare manifestation of AOSD. Here, we present a case of 22-year-old woman with no significant medical history who initially came with fever, arthralgias, myalgias, generalized weakness, and sore throat. Laboratory data showed mildly elevated transaminases and markedly elevated ferritin levels. She was diagnosed with AOSD based on Yamaguchi diagnostic criteria and was started on prednisone. Three months later, while she was on tapering dose of steroid, she presented with fever, abdominal pain, jaundice, and markedly elevated transaminases. Extensive workup excluded all potential causes of liver failure. She was diagnosed with AOSD associated acute liver failure (ALF). Intravenous (IV) methylprednisolone pulse therapy was started, with dramatic improvement in liver function. Our case demonstrated that ALF can present as a complication of AOSD and IV mega dose pulse methylprednisolone therapy can be employed as a first-line treatment in AOSD associated ALF with favorable outcome.

## 1. Introduction

Adult onset Still's disease (AOSD) is a rare systemic inflammatory disorder with a typical evanescent salmon-pink nonpruritic maculopapular rash, leukocytosis (≥10,000 WBC/mm^3^) with at least 80% neutrophils, fever, and arthralgias/arthritis [1]. Other common symptoms include sore throat, myalgias, lymphadenopathy, hepatomegaly, splenomegaly, and abdominal pain. Markedly elevated serum ferritin levels have been frequently seen. AOSD is a clinical diagnosis and several sets of classification criteria have been proposed to aid in the diagnosis. The most widely validated criteria cited in the literature are Yamaguchi's criteria, with five or more criteria of which presence of two or more major criteria have a sensitivity and specificity of 96.2% and 92.1%, respectively [2]. Hepatic involvement is frequently observed in the course of AOSD. Mild elevation in transaminases is common. Acute liver failure (ALF) is a rare manifestation, occasionally requiring urgent liver transplantation [3–9]. We report a case of ALF in a patient with recently diagnosed AOSD who was successfully treated with IV pulse methylprednisolone therapy.

## 2. Case Report

A 22-year-old African American female with a past medical history significant for AOSD presented with fever, arthritis, and abdominal pain. Three months ago, she presented with fever, arthralgia, myalgias, generalized weakness, sore throat, maculopapular skin rash, and cervical and axillary lymphadenopathy. Laboratory data showed mildly elevated transaminases and markedly elevated ferritin levels. After extensive work up including negative HIV and other acute viral illness and normal bone marrow biopsy, patient was diagnosed with AOSD based on Yamaguchi diagnostic criteria. She had met three major and four minor criteria. She was discharged on prednisone 20 mg/day. Currently, while on tapering dose of prednisone, she presented with fever, arthritis, and abdominal pain. On examination, she was slightly drowsy and was noted to have fever of 101.6°F, mild conjunctival pallor, and icteric sclera. Her abdominal examination showed epigastric and right upper quadrant tenderness. Laboratory findings included normal basic metabolic panel. Complete blood count showed leucocyte count of 4.2 × 10^9^/L, hemoglobin 11.4 gm/dL, platelet count 144 × 10^9^/L. Liver function tests showed total bilirubin of 5.4 mg/dL, aspartate aminotransferase (AST) of 4,974 U/L, alanine aminotransferase (ALT) of 2,522 U/L, alkaline phosphatase of 211 U/L, gamma glutamate transpeptidase (GGT) of 155 U/L, and albumin of 3 g/dL. Coagulation studies were prothrombin time (PT) of 18.1 seconds (151% of normal), international normalised ratio (INR) of 1.53, and activated partial thromboplastin time (APTT) of 29.1 seconds (116% of normal). Serum ferritin level was >15,000 ng/mL (normal: 40–200 ng/mL). Serum and urine toxicology screen was negative. Autoimmune workup including antinuclear antibody, rheumatoid factor, anti-mitochondrial antibody, anti-smooth muscle antibody, anti-liver/kidney microsomal antibody, immunoglobulins, ceruloplasmin, and alpha 1 antitrypsin were all negative. Serology for viral hepatitis A, hepatitis B, hepatitis C, hepatitis D, hepatitis E, herpes simplex virus (HSV), Epstein-Barr virus, Cytomegalovirus, human immunodeficiency virus (HIV), West Nile virus,* Leptospira*,* Borrelia*, and Q fever were negative. Ultrasound of the abdomen showed normal liver morphology with normal echogenicity. She was diagnosed with ALF secondary to AOSD. She was promptly started on IV pulse methylprednisolone therapy 1 gram/day for 3 days. After 3 days of treatment with pulse methylprednisolone therapy, her liver enzymes began to trend down dramatically. IV steroids were then switched to peroral prednisone 40 mg/day. She was discharged in a stable condition with near normal liver function tests after 8 days of hospitalization. One year later, she remained in remission on low dose prednisone.

## 3. Discussion

Still's disease is named after George. F. Still, who originally described 22 cases of chronic polyarthritis usually referred to as juvenile rheumatoid arthritis in children in 1897. In 1971, Eric Bywaters described 14 cases resembling Still's disease that started in adult life, hence the name adult onset Still's disease [10]. In a retrospective study of 62 patients in West France, the estimated incidence of AOSD was 0.16 per 100000 inhabitants. Mean age of the study population was 36 [11]. Based on an epidemiological survey conducted in Japan, estimated crude prevalence was calculated as 0.73 and 1.47 per 100,000 population for males and females, respectively, with a female to male ratio of 2 : 1 [12].

The etiology of AOSD remains unknown. AOSD is considered as multisystemic disorder in which several cytokines including interleukin (IL), mainly IL-1, IL-6, and IL-18, interferon (IFN) gamma, and tumor necrosis factor (TNF) alpha have been implicated in the pathogenesis [1]. IL-18 has been identified to play a key role in AOSD pathogenesis including high serum ferritin levels and liver injury in AOSD [8].

Clinical course of AOSD is usually benign. Rarely, serious complications such as ALF, macrophage activation syndrome/hemophagocytic syndrome, pericarditis, cardiac tamponade, disseminated intravascular coagulation, serous peritonitis, pleuritis, and respiratory failure are seen [1]. Our patient presented with ALF.

Hepatic involvement usually manifesting as asymptomatic elevation in transaminases with or without hepatomegaly is frequently seen in AOSD; indeed liver dysfunction is one of Yamaguchi's minor criteria. ALF is a rare complication of AOSD. ALF can occur at the time of AOSD diagnosis, during tapering of immunosuppressive therapy, or years after diagnosis when other symptoms of AOSD are well controlled. After extensive review of literature, seventeen cases of ALF in AOSD were reported [3–9, 13–21], of which seven (41.1%) required liver transplantation [3–9]. Characteristics of previously published cases of ALF in AOSD are highlighted in Table 1.

Of the seventeen cases reported, twelve patients (12/17 = 70.5%) are females, as our patient [3, 5, 7–9, 13–15, 17–19]. Seven patients had AOSD diagnosed at the time of ALF presentation (7/17 = 41.1%) [3, 9, 14, 15, 18–20]. Eight patients (8/17 = 47%) had AOSD diagnosed prior to the onset of ALF (timeline between AOSD diagnosis and onset of liver failure ranging from 10 days to 3 years) [5–8, 13, 16, 17]. One patient (1/17 = 5.88%) had AOSD diagnosed following liver transplantation for ALF when symptoms of the disease recurred with tapering of oral steroids and in one patient (1/17 = 5.88%) we were unable to obtain information regarding the timeline of AOSD diagnosis and onset of ALF [4, 21]. Eight patients (8/17 = 47%) were on steroids with or without combination with other immunosuppressant drugs for the treatment of AOSD prior to onset of ALF [5, 7, 8, 13, 14, 16, 17]. Of the eight patients with prior AOSD diagnosis, four received treatment with methylprednisolone pulse therapy for ALF [5–8]. All four patients subsequently received liver transplantation and recovered with uneventful follow-up. Two patients recovered with pulse dexamethasone therapy combined with cyclosporine and one patient recovered with prednisolone and anakinra without the need for liver transplantation [16, 17]. One patient was on prednisolone at the time of ALF and went into grade IV hepatic encephalopathy the next day and died [13]. Of the seven patients who were diagnosed with AOSD simultaneously at the time of presentation with ALF, two patients received therapy with pulse methylprednisolone; of them, one patient recovered and the other required liver transplant and died after transplantation from disseminated intravascular coagulation and intraventricular hemorrhage [9, 15]. Three patients were started on prednisolone (not pulse therapy) and all of them recovered [19–21]. One patient was treated with ursodeoxycholic acid and recovered [14]. One patient did not receive any steroids and underwent liver transplantation [3].

Based on the above data it is evident that patients with ALF who were simultaneously diagnosed with AOSD responded to steroid therapy with or without combination with other immunosuppressants (4 patients out of 7 patients, 57.14%) better than those who had prior diagnosis of AOSD and were already on maintenance immunosuppressant therapy (3 patients out of 8, 37.5%) [15–20]. Also it is noted that response to pulse steroid therapy decreases with increase in the number of AOSD exacerbations presenting with ALF [7, 8]. In our patient, this is the first exacerbation of AOSD presenting with ALF for which we promptly started IV pulse methylprednisolone with a good outcome. To our knowledge, this is the first case reported in an African American female patient with prior AOSD diagnosis presenting with ALF who responded to IV pulse methylprednisolone without the need for liver transplantation.

Liver biopsy findings in active AOSD are nonspecific which can include periportal inflammatory infiltrates, Kupffer cell hyperplasia, periportal fibrosis, and focal hepatitis with submassive or massive necrosis. The utility of liver biopsy in the diagnosis of AOSD is undetermined. It may be useful in identifying previously undiagnosed concomitant liver diseases, which may be exacerbated by steroids and immunosuppressive therapies for adult onset Still's disease, which can influence the management and eventual outcome of liver injury in AOSD [22].

Liver dysfunction in AOSD often reflects underlying disease activity [23]. Improvement in liver function occurs concomitantly with recovery of AOSD flare following initiation of appropriate therapy. Steroids are usually employed as the first-line agents for AOSD associated ALF. Intravenous methylprednisolone pulse therapy is recommended in patients with acute multisystem flares involving vital organs such as cardiac tamponade, pleuritis, respiratory failure, ALF, severe anemia due to macrophage activation syndrome (MAS), disseminated intravascular coagulation, and serous peritonitis [24]. As described in our patient, remarkable improvement in liver enzymes occurred following three days of IV pulse methylprednisolone therapy (1 gram/day). Steroid-refractory ALF cases secondary to AOSD have showed response to treatment with cyclosporine and IL-1 receptor antagonist (anakinra) [16, 17]. Occasionally, ALF persists despite aggressive medical therapy and expeditious evaluation for liver transplantation is warranted under such circumstances.

Our case demonstrated that ALF can be a rare presentation of AOSD in the absence of other potential causes of liver failure. Prompt initiation of IV mega dose methylprednisolone pulse therapy may be indicated as a first-line treatment in AOSD associated ALF with favorable outcome.

## Figures and Tables

**Table 1:** Characteristics of cases reported on ALF in Adult Onset Still's Disease (AOSD).

Author [Reference]	Gender/Age (Years)	Interval between AOSD diagnosis and ALF	Hepatotoxics prior to the onset of ALF	Baseline AOSD treatment before ALF	Treatment of ALF associated with AOSD	Underwent liver transplantation	Outcome
Liese et al. [3]	F/24	Simultaneous	None	None	Extracorporeal liver support	Yes	Recovered
Terán et al. [4]	M/23	AOSD diagnosed after ALF when symptoms recurred	None	None	Supportive care	Yes	Recovered
Taccone et al. [5]	F/28	1 month	None	Prednisolone 8 mg/day	IV Methylprednisolone 500 mg and MARS	Yes	Recovered
Mc Farlane et al. [6]	M/21	<1 month	Acetaminophen	Aspirin, Acetaminophen,	IV one pulse of Methyprednisolone 250 mg and then 20 mg IV BID	Yes	Recovered
Yamanaka et al. [7]	F/20	3 years	None	Cyclosporine, Prednisolone 15 mg/day	IV Methylprednisolone pulse 500 mg/day for 3 days	Yes	Recovered
Ogata et al. [8]	F/20	3 years	None	Prednisolone 15 mg/day	2 days of pulse IV Methyl prednisolone 1 gm per day and then plasma exchange	Yes	Recovered
Dino et al. [9]	F/44	Simultaneous	Aspirin 1.8 gram/day	None	IV MethylPrednisolone 1 gm/day for 2 days	Yes	Died
Thabah et al. [13]	F/29	10 days	Indomethacin	PO Prednisolone 40 mg/day and indomethacin	Steroids	No	Died
Ott et al. [14]	F/25	Simultaneous	None	Prednisolone 16 mg/day for an undetermined rheumatic disease	UDCA	No	Recovered
Linde et al. [15]	F/39	Simultaneous	Acetaminophen, Ibuprofen	NSAIDs	3 days of IV Methyprednisolone 250 mg per day	No	Recovered
Mylona et al. [16]	M/46	2 months	None	Tapering dose of Prednisolone	Prednisolone 75 mg/day PO and Anakinra 100 mg/day PO	No	Recovered
Nagashima et al. [17]	F/51	3 years	Indomethacin	Oral betamethasone, methotrexate and Indomethacin	IV Pulse dexamethasone 100 mg/day and Cyclosporine 120 mg/day	No	Recovered
	F/32	2 weeks	None	Prednisolone 50 mg/day and Cyclosporine 150 mg/day	3 days of IV dexamethasone pulse therapy 120 mg/day, dexamethasone 5 mg/day cyclosporine	No	Recovered
Hogan et al. [18]	F/35	Simultaneous	None	Sulfasalazine for rheumatic disease	2 days of Methylprednisolone 80 mg IV every 8 hrs	No	Recovered
Takami et al. [19]	F/74	Simultaneous	None	None	Prednisolone 30 mg/day	No	Recovered
Schuster et al. [20]	*50 *y	Simultaneous	None	None	Prednisolone, methotrexate	No	Recovered
Atsukawa et al. [21]	34	NM	None	None	Prednisolone 40 mg/day	No	Recovered

Gm/dL-grams/deciliter, IV-Intravenous, PO-peroral, MARS-Molecular Adsorbent Recirculating System, mg-milligrams, BID-twice a day, UDCA-Ursodeoxycholic acid, NSAIDS-Non steroidal anti inflammatory drugs, hrs-hours, NM-Not mentioned.
